# Repression of the *PRELP* gene is relieved by histone deacetylase inhibitors through acetylation of histone H2B lysine 5 in bladder cancer

**DOI:** 10.1186/s13148-022-01370-z

**Published:** 2022-11-12

**Authors:** Kanto Shozu, Syuzo Kaneko, Norio Shinkai, Ai Dozen, Hirofumi Kosuge, Makoto Nakakido, Hidenori Machino, Ken Takasawa, Ken Asada, Masaaki Komatsu, Kouhei Tsumoto, Shin-Ichi Ohnuma, Ryuji Hamamoto

**Affiliations:** 1 Division of Medical AI Research and Development, National Cancer Center Research Institute, 5-1-1 Tsukiji, Chuo-Ku, Tokyo, 104-0045 Japan; 2 Department of Obstetrics and Gynecology, University of Toyama, Toyama, Japan; 3 RIKEN Center for Advanced Intelligence Project, Cancer Translational Research Team, Tokyo, Japan; 4 Department of NCC Cancer Science, Biomedical Science and Engineering Track, Graduate School of Medical and Dental Sciences, Tokyo Medical and Dental University, Tokyo, Japan; 5 School of Engineering, The University of Tokyo, Tokyo, Japan; 6 UCL Institute of Ophthalmology, University College London, 11-43 Bath Street, London, EC1V 9EL UK; 7 Department of Oncology, The Hutchison/MRC Research Center, University of Cambridge, Hills Road, Cambridge, CB2 2XZ UK

**Keywords:** Gene expression, Extracellular matrix proteins, PRELP, HDACi, H2BK5ac, Bladder cancer

## Abstract

**Background:**

Proline/arginine-rich end leucine-rich repeat protein (PRELP) is a member of the small leucine-rich proteoglycan family of extracellular matrix proteins, which is markedly suppressed in the majority of early-stage epithelial cancers and plays a role in regulating the epithelial–mesenchymal transition by altering cell–cell adhesion. Although PRELP is an important factor in the development and progression of bladder cancer, the mechanism of *PRELP* gene repression remains unclear.

**Results:**

Here, we show that repression of *PRELP* mRNA expression in bladder cancer cells is alleviated by HDAC inhibitors (HDACi) through histone acetylation. Using ChIP-qPCR analysis, we found that acetylation of lysine residue 5 of histone H2B in the *PRELP* gene promoter region is a marker for the de-repression of PRELP expression.

**Conclusions:**

These results suggest a mechanism through which HDACi may partially regulate the function of PRELP to suppress the development and progression of bladder cancer. Some HDACi are already in clinical use, and the findings of this study provide a mechanistic basis for further investigation of HDACi-based therapeutic strategies.

**Supplementary Information:**

The online version contains supplementary material available at 10.1186/s13148-022-01370-z.

## Background

Although cancer is often thought to be a disease caused by mutations in genes involved in growth and differentiation [1, 2], epigenetic changes that alter the structure of chromatin and consequently affect gene transcription can also occur at any stage during cancer progression [3, 4]. Chromatin consists of a complex of DNA and a series of histones in the nucleus of eukaryotic cells, and its basic unit is called a nucleosome. The nucleosome consists of 147 bp of DNA wrapped around an octamer of four core histones H2A, H2B, H3, and H4 [5, 6]. The N-terminal tail of the core histones contains lysine and arginine residues, rendering them susceptible to posttranslational modifications. Among the modifications, acetylation of core histones neutralizes the positive charge of lysine residues, thereby weakening their interaction with negatively charged DNA molecules. Changes in acetylation are particularly dynamic and reversible mechanisms that are altered by a variety of stimuli [7]. Specifically, the transition from one state to another is catalyzed by histone acetyltransferases (HATs) and histone deacetylases (HDACs). HATs can be divided into three major families, whereas the HDACs are grouped into four families consisting of 18 different HDACs [8]. Currently, there is a growing interest in HDACi as potent anticancer therapeutics. In particular, the application of HDACi has been shown to be useful in hematological diseases [9]. For example, in cutaneous T cell lymphoma (CTCL), the dynamic chromatin architecture of CTCL explains the efficacy of monotherapy with HDACi [10]. In contrast, monotherapy for solid tumors has been shown to be largely ineffective, and therefore the focus has been directed toward combined inhibition strategies [11].

Bladder cancer is one of the most common cancers worldwide, accounting for over 500,000 new cases and 200,000 cancer-related deaths annually [12]. Low-grade non-muscle-invasive bladder cancer (NMIBC) rarely acquires invasive features but usually has the potential to recur and a 5-year survival rate of approximately 90%. In contrast, high-grade muscle-invasive bladder cancer (MIBC; stage T2 or higher) often progresses to metastatic cancer and has a poor prognosis, with a 5-year survival rate of < 50% [13, 14]. In terms of genomic aberrations, the tumors are usually resistant to various therapeutic regimens because of the high frequency of somatic mutations and high molecular heterogeneity. Because chromatin-regulated genes are more frequently mutated in MIBC than in other epithelial tumors [15], targeted therapies for chromatin abnormalities in chemo-resistant clones may prove beneficial for this disease. To date, methotrexate–vinblastine–adriamycin–cisplatin and gemcitabine–cisplatin have been the backbone of systemic chemotherapy. However, despite initial chemosensitivity, the majority of treated patients eventually develop chemoresistance, resulting in significantly shortened survival [14]. Therefore, there is an urgent need to develop new systemic strategies for the clinical management of this disease.

Small leucine-rich proteoglycans (SLRPs) constitute a family of 17 proteoglycans that are secreted as extracellular matrix (ECM) proteins [16]. Members of SLRPs not only modify ECM tissues but also function as regulators of ligand-induced signaling pathways [16–19]. We previously showed that the expression levels of two SLRPs (secreted ECMs), osteomodulin (OMD) and PRELP, are strongly repressed in the majority of early-stage epithelial cancers and that they play a role in the regulation of epithelial–mesenchymal transition (EMT) by altering cell–cell adhesion [20]. Furthermore, they were shown to be important factors that negatively regulate the development and progression of bladder cancer [20]. Although we showed that chromosome 9q deletion, which is involved in the development of bladder cancer, is responsible for the loss of function of the OMD gene, the mechanism of *PRELP* gene repression remains unresolved.

In this study, we showed that *PRELP* gene repression is relieved by HDACi mediated by histone acetylation in bladder cancer cells. In addition, we showed that acetylation of lysine residue 5 of histone H2B (H2BK5ac) in the *PRELP* gene promoter region is a marker that relieves *PRELP* gene repression. These results provide mechanistic insights into HDACi-mediated inhibition of the development and progression of bladder cancer, partly via regulation of PRELP. Some of these inhibitors are already in clinical application, and our data provide a mechanistic basis for considering their action as a therapeutic option.

## Results

### Mechanism of PRELP repression is not mainly mediated by a genetic mutation, copy number aberration (CNA), or DNA methylation

**Fig. 1:**
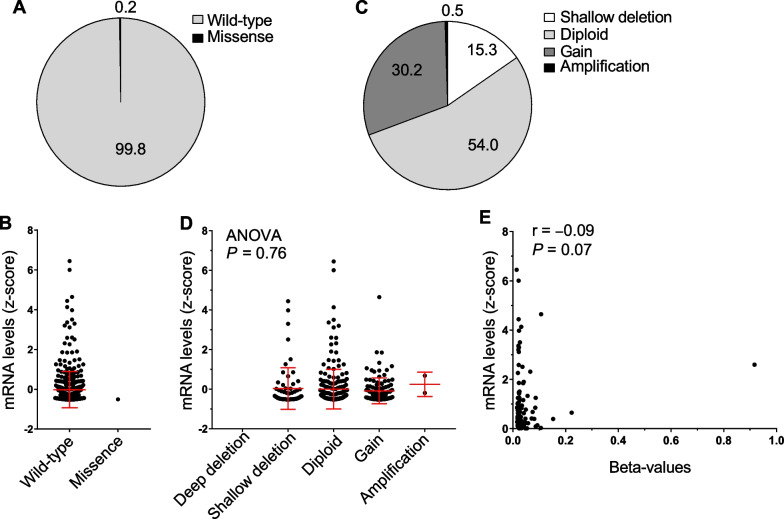
Correlation between *PRELP* gene expression and genetic mutation, copy number alteration (CNA), and DNA methylation in bladder cancer. **A** Pie chart showing the percentage of missense mutations in the *PRELP* gene (*n* = 408). Wild type is shown in gray and missense mutations are shown in black. **B** *PRELP* mRNA expression (*z*-scores relative to diploid samples; *y*-axis) are plotted against a missense mutation (*n* = 1) or wild type (*n* = 407; *x*-axis). **C** Pie chart showing CNAs in *PRELP* gene (*n* = 404). Shallow deletion (CNA = − 1), diploid (CNA = 0), gain (CNA =  + 1) and amplification (CNA =  + 2) are shown in white, light gray, gray, and black, respectively. **D** Same as **B**, but plotted by distinct type of CNAs (*x*-axis); deep deletion (*n* = 0), shallow deletion (*n* = 62), diploid (*n* = 218), gain (*n* = 122), and amplification (*n* = 2). **E** Same as **B**, but plotted against DNA methylation level (beta-values; *x*-axis) of *PRELP* (*n* = 408). Spearman correlation coefficient (*r*) between DNA methylation and *PRELP* mRNA expression is shown

Previously, we showed that the mRNA expression of *PRELP* was strongly repressed in the majority of epithelial cancers [20]. To investigate the association between *PRELP* mRNA expression and genomic aberrations, we first analyzed the correlation between *PRELP* expression and somatic mutations and CNAs using a comprehensive genomic dataset of 412 MIBCs characterized in multiple TCGA platforms [21]. In 99.8% of the cases (407/408), PRELP retained its wild-type form, and there was no association between somatic mutations and *PRELP* mRNA expression (Fig. 1A, B). Deletions in the *PRELP* gene were found in 15.3% of cases, and *PRELP* gene amplification was found in 30.7% of cases. However, these alterations did not correlate with *PRELP* mRNA expression (Fig. 1C, D). These results indicate that somatic mutations and CNAs are not strongly associated with *PRELP* mRNA expression. We next examined the DNA methylation status of the *PRELP* gene region that has been suggested to be associated with gene silencing but found no apparent association (Fig. 1E). These results suggest that the repression of *PRELP* mRNA expression is not dependent on somatic mutations, CNAs, or DNA methylation, but rather on transcriptional regulatory mechanisms associated with protein posttranslational modifications, such as histone modification.

### Elucidation of the mechanism of repression of *PRELP* gene using in vitro models of bladder cancer

**Fig. 2:**
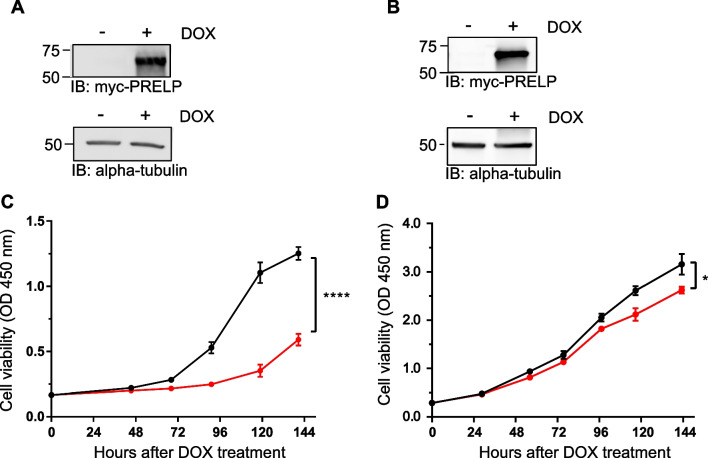
Reduced cell viability associated with induced *PRELP* gene expression in bladder cancer cells. *PRELP* gene expression was induced by the addition of doxycycline (DOX, 1 μg/ml) in the lentiviral expression system. Expression of myc-tagged PRELP protein was analyzed using whole-cell extracts from RT4 (**A**) and J82 (**B**) cells with or without DOX. The left side indicated the protein size marker. Alpha-tubulin; loading control. Cell viability of RT4 (**C**) and J82 (**D**) cells was evaluated using cell counting kit (CCK)-8 assays. Black lines; without DOX, Red lines; with DOX. Error bars indicate biological replicates (*n* = 3). Statistical analysis was performed using paired Student’s *t* test. *P < 0.05, ****P < 0.0001

Next, we used two bladder cancer cell lines, RT4 and J82 to investigate the relationship between histone modification and repression of *PRELP* gene expression [22–24]. As we have already shown that RT4 and J82 cell lines have significantly reduced mRNA expression of *PRELP* compared to normal tissues [20], they constitute suitable cell lines for histone modification-related functional analysis. To further investigate whether these cell lines faithfully reflect the antitumor effects of PRELP [20], we stably introduced the *PRELP* gene into these cells using a lentivirus. The transduced *PRELP* gene contained an inducible promoter (Tet-On system), which allowed us to control the timing of *PRELP* gene expression. Therefore, we added doxycycline to the established cell lines to induce PRELP protein expression. Western blotting results showed that PRELP protein was overexpressed following the addition of doxycycline (Fig. 2A, B). Importantly, we observed that cell proliferation was significantly suppressed in the cell lines in which PRELP protein was induced (Fig. 2C, D), confirming that PRELP protein functions negatively in cell proliferation [20]. These results suggest that both RT4 and J82 cell lines are suitable for the analysis of PRELP protein expression status and function.

### HDACi reverses the repression of *PRELP* gene expression

**Fig. 3:**
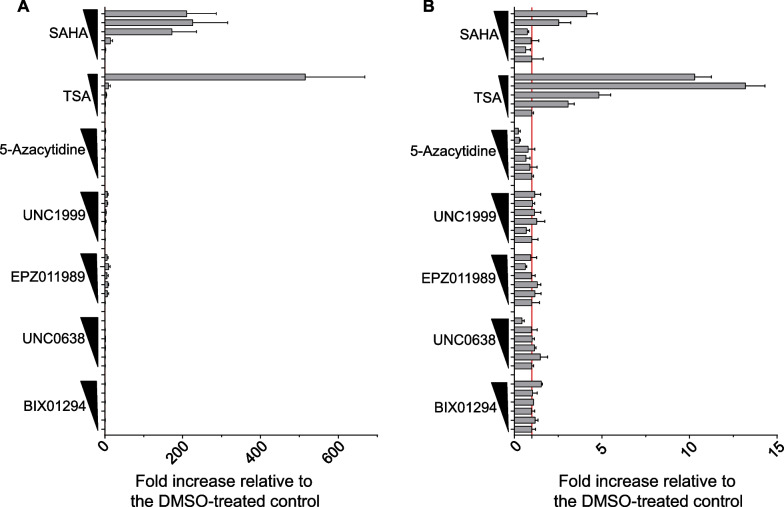
Restoration of *PRELP* gene expression by HDAC inhibitors in bladder cancer cells. RT-qPCR analyses of *PRELP* (gray bars) gene expression in RT4 (**A**) and J82 cells (**B**). The cells were treated with either DMSO or increasing concentration of BIX01294 (0, 0.625, 1.25, 2.5, 5, and 10 μM), UNC0638 (0, 0.625, 1.25, 2.5, 5, and 10 μM), EPZ011989 (0, 0.625, 1.25, 2.5, 5, and 10 μM), UNC1999 (0, 0.625, 1.25, 2.5, 5, and 10 μM), 5-azacytidine (0, 0.625, 1.25, 2.5, 5, and 10 μM), TSA (0, 0.25, 0.5, 1.0, and 2 μM), and SAHA (0, 0.3125, 0.625, 1.25, 2.5, and 5 μM) for 72 h. Data are presented as the mean ± standard deviation (SD) of three technical replicates. The *x*-axis shows the fold increase relative to DMSO-treated control. The *y*-axis shows the name and concentration of each inhibitor. The red line indicates a fold increase of 1

To investigate the relationship between histone modification and repression of *PRELP* gene expression, we treated RT4 and J82 cells with several inhibitors of histone modification-related enzymes and then analyzed *PRELP* mRNA expression by RT-PCR. The inhibitors used were (1) G9a/GLP inhibitors, BIX01294 and UNC0638, which are compounds that selectively inhibit an enzyme that methylates histone H3 lysine 9 and are known to negatively regulate transcription [25, 26]; (2) Ezh2 and Ezh1/Ezh2 inhibitors: EPZ011989 and UNC1999, which are compounds that selectively inhibit enzymes that add up to three methyl groups on lysine 27 of histone H3, particularly H3K27me3, the most important epi-marker for cancer diseases [27, 28]. None of the compounds promoted increased *PRELP* mRNA expression in RT4 cells (Fig. 3A). Furthermore, consistent with the TCGA database analysis (Fig. 1E), treatment with 5-azacytidine, an inhibitor of DNA methyltransferase 1 (DNMT1) [29, 30], did not show consistent upregulation of *PRELP* mRNA expression (Fig. 3A). Finally, we used the pan-HDACi, trichostatin A (TSA), which inhibits histone deacetylation [31, 32]. Strikingly, there was a marked increase in *PRELP* mRNA expression in RT4 cells (Fig. 3A). To further verify these results, we confirmed the *PRELP* mRNA expression by RT-PCR after treatment with another pan-HDACi, suberanilohydroxamic acid (SAHA), which is used clinically as an anticancer drug [33, 34]. Indeed, there was a marked increase in the mRNA expression of *PRELP* following treatment with SAHA (Fig. 3A). Similar results were obtained in J82 cells (Fig. 3B). In summary, these results suggest that the repression of *PRELP* gene expression involves protein deacetylation and that HDACi reverse this repression.

### Repression of *PRELP* mRNA expression is mediated by deacetylation of H2BK5

**Fig. 4:**
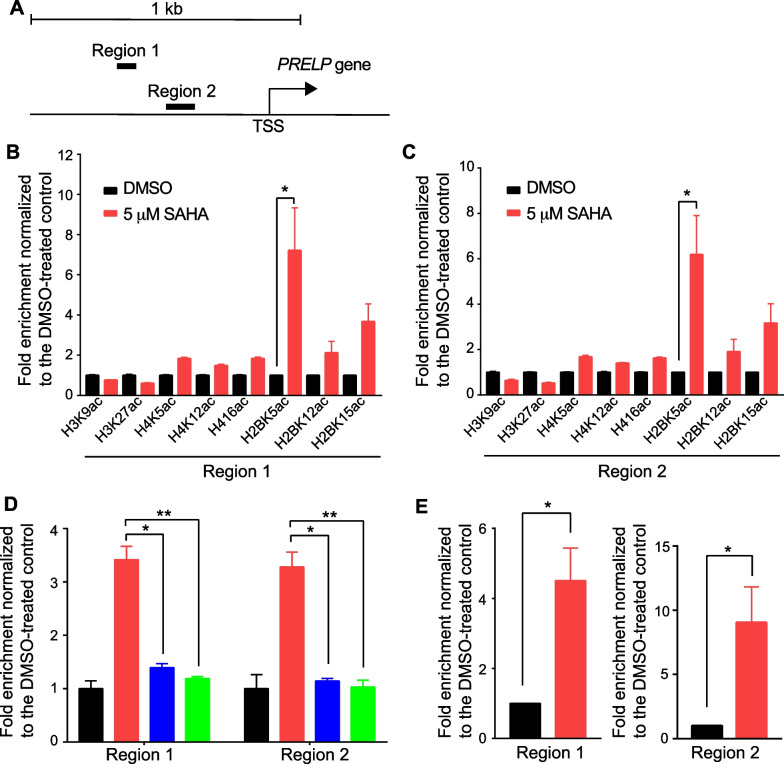
The HDAC inhibitor, SAHA, restores the expression of the *PRELP* gene through H2B acetylation at the promoter regions in the bladder cancer cells. **A** Genomic positions (Region 1 and Region 2) of the two PCR-amplified DNAs fragments are shown. **B** ChIP-qPCR was performed using various histone antibodies at the *PRELP* gene promoter region (Region 1) in RT4 cells treated with DMSO or 5 μM SAHA for 24 h. **C** Same as (**B**), except that region 2 was similarly analyzed. **D** The acetylation status of H2BK5 in the promoter region (Region 1 and Region 2) of *PRELP* gene in RT4 cells treated with two inhibitors, C646 (5 μM), SGC-CBP30 (5 μM), in the presence of SAHA (5 μM). Black; DMSO, Red; SAHA, Blue; SAHA with C646, Green; SAHA with SGC-CBP30. **E** H2BK5ac ChIP-qPCR in J82 cells. The ChIP enrichment (red bars) is normalized against the values obtained with the same antibodies in DMSO-treated control cells (black bars). Bars represent the mean ± SD of three technical replicates or mean ± standard error of the mean (SEM) of three biological replicates. Statistical analysis was performed using paired Student’s *t* test. *P < 0.05, **P < 0.01

The regulatory mechanism of gene expression involving protein acetylation and deacetylation is often regulated by modifications of the histone in the gene promoter region [7]. To investigate the acetylation/deacetylation status of histones in the *PRELP* gene promoter region, we performed ChIP followed by quantitative PCR (ChIP-qPCR) [35, 36]. Chromatin was immunoprecipitated using histone acetyl group-specific antibodies. The DNA bound to the immunoprecipitated chromatin was purified, and PCR primers specific to the *PRELP* gene promoter region (Fig. 4A) were used for PCR amplification. The antibodies used in this study were specific to acetyllysine residues 9 and 27 of histone H3, acetyllysine residues 12 and 16 of histone H4, and acetyllysine residues 5, 12, and 15 of histone H2B (Fig. 4B). Although no increase in acetylation of histone H3 and H4 was observed following SAHA treatment, we found a noticeable increase in the acetylation of H2B. In particular, H2BK5ac expression was significantly increased in RT4 cells (Fig. 4B). Furthermore, ChIP-qPCR experiments using different PCR primer sets designed for the *PRELP* gene promoter region (Fig. 4A) as well as gene body region (Additional file 1: Fig. S1) showed a significant increase in H2BK5ac following SAHA treatment (Fig. 4C, Additional file 1: Fig. S1). Considering histone acetyltransferases (HATs) as transcriptional activators, we next examined whether the p300/CBP protein complex might be involved in the transcriptional activation of *PRELP* via H2BK5 acetylation. To this end, RT4 cells were treated with p300/CBP inhibitors along with SAHA addition. Indeed, ChIP-PCR experiments showed that H2BK5 acetylation by SAHA was diminished by adding histone acetyltransferase inhibitors, C646 and SGC-CBP30 (Fig. 4D). These results suggest that p300/CBP can acetylate H2BK5 in the promoter region of the *PRELP* gene. Although not statistically significant, acetylation of H2BK15 was also observed (see Fig. 4B, C). These results are consistent with CBP/p300 being involved in the acetylation of H2B, including lysine 15 [37]. H2BK5ac was also significantly increased in the *PRELP* gene promoter region in J82 cells after SAHA treatment (Fig. 4E). Although H2BK5 acetylation was increased in the protein as a whole following SAHA treatment (Additional file 1: Fig. S2), our results indicate that repression of *PRELP* gene expression involves deacetylation of H2BK5ac in the promoter region, and SAHA treatment reverses the repression via H2BK5ac in bladder cancer cells.

### Antitumor effects of HDACi in combination with cisplatin on bladder cancer cells

**Fig. 5:**
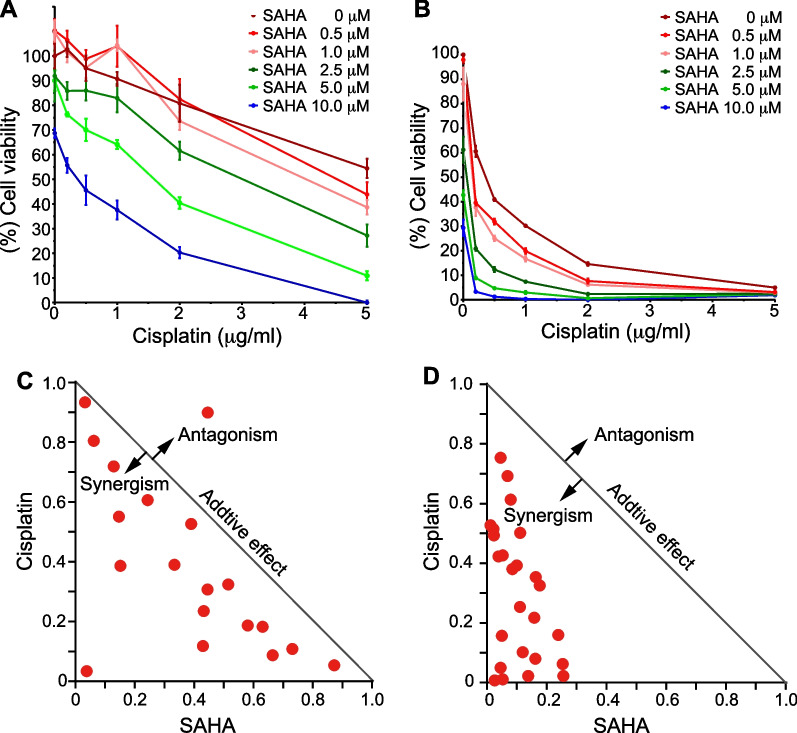
Combinatory effect of cisplatin and SAHA on viability of bladder cancer cells. Cells were treated with cisplatin alone or in combination with SAHA. Cisplatin and SAHA were used at a concentration range of 0, 0.2, 0.5, 1.0, 2.0, 5.0 μg/ml and 0, 0.5, 1.0, 2.5, 5.0, 10.0 μM, respectively. Cell viability of RT4 (**A**) and J82 (**B**) cells was evaluated using cell counting kit (CCK)-8 assays. We set the viability of cells with no inhibitor as 100% (*n* = 4). *y*-axis shows cell viability (%), and *x*-axis shows the concentration of cisplatin. Normalized isobologram of RT4 (**C**) and J82 (**D**) show cisplatin and SAHA synergism as described in “Methods”

As HDACi have been shown to have very little effect on solid tumors as single agents, combinatorial use of inhibitors has attracted considerable attention [11]. Therefore, we investigated the antitumor effects of cisplatin in combination with SAHA, an FDA-approved anticancer drug that has been shown to enhance *PRELP* gene expression as described above. The results showed that combination therapy with cisplatin and SAHA severely inhibited the growth of RT4 and J82 cells compared to either treatment alone (Fig. 5A, B). When data were analyzed based on the combination index (CI), strong synergism existed with CI less than 1 for dose combinations tested (Fig. 5C, D), consistent with the synergistic effect of cisplatin and TSA [38]. In particular, when SAHA (at a concentration of 2.5 μM or higher) was combined with cisplatin, the anticancer effect of cisplatin at lower concentrations was significantly enhanced (Fig. 5A, B). This is consistent with the fact that the *PRELP* gene is derepressed at this concentration range and exerts an antitumor effect (Figs. 2C, D, 3A, B). It would be interesting to gain further insights into other HDACi that are currently under clinical trials. Among them, entinostat, an inhibitor of class I HDACs, exhibited a marked increase in *PRELP* mRNA expression (Additional file 1: Fig. S3) and a strong synergistic effect with cisplatin in RT4 and J82 cells (Additional file 1: Fig. S4) [39]. Although these results suggest that Class I HDACs are involved in suppressing PRELP expression, we further tested various selective HDAC inhibitors to understand the selectivity of HDACs for *PRELP* gene repression (Additional file 1: Fig. S5). Indeed, the selective class I HDAC inhibitor, tacedinaline, showed an increase in *PRELP* expression, albeit less pronounced in J82, indicating that class I HDACs contribute to the inhibition of *PRELP* expression. On the other hand, Santacruzamate A, a selective HDAC2 inhibitor, did not upregulate *PRELP* expression. Somewhat unexpectedly, we found the marked upregulation of *PRELP* expression in LMK-235, a selective HDAC4,5 inhibitor. Given that LMK-235 is known to inhibit HDACs other than HDAC4,5 with a relatively higher concentration [40], it is unclear whether or not HDAC4,5 are involved with *PRELP* repression. These results suggest that HDAC1 is likely involved in the inhibition of PRELP expression, but further verification is needed because other HDACs may also be involved in the inhibition of PRELP expression. In sum, these results suggest that HDACi, which activate *PRELP* gene expression, enhance the inhibition of cell proliferation when combined with cisplatin. Of note, paclitaxel, a different class of chemotherapeutic agent, did not show synergistic anticancer activity with SAHA in RT4 and J82 cells (Additional file 1: Fig. S6).

## Discussion

HDACs are aberrantly expressed in various tumors. For example, class I HDACs have been found to be overexpressed in bladder tumors [41], breast tumors [42], prostate tumors [43], and renal cells [44], and overexpression of HDAC2 and HDAC3 has also been shown to be associated with clinicopathological indicators of disease progression [42]. Many studies have reported the anticancer effects of HDAC inhibition with the induction of apoptotic cell death [45]. In particular, the synergistic anticancer effect of combination therapy with HDACi and other drugs has already been demonstrated in several carcinomas [11], and different mechanisms of action have also been reported for each type of carcinoma [46]. The mechanism of action of this synergistic effect is complex, and various scenarios are possible. For example, each mechanism of action may prevent the acquisition of drug resistance by acting on different pathways [9]. However, each mechanism of action may work in a complementary manner to elicit a robust anticancer effect [47].

This study presents an antitumor mechanism for HDACi, which are often thought to globally enhance gene transcription because they increase overall histone acetylation (Additional file 1: Fig. S2); however, approximately half of the genes with variable expression are negatively regulated, likely through the functions of non-histone proteins [48]. However, in the case of the *PRELP* gene, HDACi positively affects *PRELP* gene expression, as it increases H2BK5 acetylation in its promoter region and activates gene transcription (Figs. 3, 4). We previously showed that *PRELP* gene overexpression inhibits cancer progression by blocking TGF-β and EGF pathways, reversing EMT, activating cell adhesion, and inhibiting various oncogenic pathways [20]. Then, do HDAC inhibitors also cause EMT reversal? Indeed, Tang et al. and Zhao et al. have identified multiple class I HDAC inhibitors that cause EMT reversal, consistent with our results of PRELP expression induced by class I HDAC inhibitors [49, 50]. The fact that PRELP expression does not affect the expression levels of HDAC1, 2 (Additional file 1: Fig. S7) is consistent with the view that the *PRELP* gene is activated following acetylation of H2BK5 and orchestrates the EMT program in bladder cancer cells. Of note, the *PRELP* gene is strongly repressed in the majority of early-stage epithelial cancers [20]. Therefore, it will be interesting to test whether HDACi alleviates the repression of *PRELP* gene expression via acetylation of H2BK5 in various other epithelial cancers.

Recent data suggest that H2BK5ac is a reliable predictor of gene expression [51] and an important modifier in the orchestration of EMT programs. Mechanistically, MAP3K4-regulated chromatin modifiers CBP and HDAC6 each regulate thousands of genes during EMT by controlling promoter acetylation of H2BK5 [52, 53]. Although the increase in acetylation levels of H2BK5 was the same in the two cell lines, the antiproliferative effects of PRELP overexpression (Fig. 2C, D) and restoration of *PRELP* gene expression by HDACi (Fig. 3A, B) were more pronounced in RT4 cells than in J82 cells. On the other hand, the combination of SAHA with cisplatin was much more effective in J82 cells than in RT4 cells (Fig. 5). These results suggest that the acetylation of H2BK5 may have different effects on open chromatinization and subsequent recruitment of transcription factors and on the phenotypic output of the cells, depending on the cell.

However, we would also like to clarify that the data presented in this study are insufficient to explain the molecular basis of these findings. First, we did not demonstrate whether the acetylation of H2BK5 is directly involved in the expression of the *PRELP* gene. Second, PRELP is a secreted ECM protein; therefore, it is unclear whether it has a direct role in the orchestration of the EMT program. Recent proteomic studies have suggested that PRELP interacts with two growth factor receptors, the insulin-like growth factor I receptor (IGFI-R) and the low-affinity nerve growth factor receptor (p75NTR) [54], and further showed that SAHA treatment enriched endogenous PRELP protein on the membrane fraction (Additional file 1: Fig. S8), supporting our hypothesis. Nevertheless, there is a need to elucidate the mechanism of the anticancer effects associated with the administration of PRELP as an ECM protein.

The clinical application of HDACi in solid tumors has been largely disappointing mainly due to limited combination chemotherapeutic studies and lack of patient stratification. In contrast, it has been reported that treatment with HDACi causes hyperacetylation of histones and relaxation of chromatin structure, leading to efficient DNA damage and cell death when treated with DNA-interacting drugs such as cisplatin. Indeed, in some preclinical and clinical settings, HDACi pretreatment approach has been reported to allow the use of lower doses of chemotherapeutic agents, consistent with the above explanation [55]. Although elevated *PRELP* gene expression during HDACi pretreatment may help determine the dose of HDACi with DNA-interacting chemotherapeutic agents needed to achieve a better therapeutic effect, further validation using animal studies is needed. Thus, activation of the *PRELP* gene with the relaxation of chromatin structure may be a good biomarker for the combination of HDACi and chemotherapy.

## Conclusions

This study revealed that HDACi promotes the acetylation of H2BK5 leading to *PRELP* mRNA expression. We also found that the acetylation of H2BK5 in the promoter region of the *PRELP* gene was associated with the restoration of *PRELP* gene expression. Thus, the activation of PRELP is an indicator of anticancer activity associated with changes in chromatin structure accompanying histone acetylation and may be a useful biomarker in combination strategies using HDACi and chemotherapy.

## Methods

### Database analysis

RNA-seq, DNA promoter methylation, DNA copy number, gene mutation, and clinical data of 412 patients with MIBC in the Cancer Genome Atlas (TCGA) cohort [21] were sourced from the cBioPortal (http://www.cbioportal.org/) for cancer genomics [56, 57]. The individual data used to generate the graphs are listed in Additional file 2: Tables S1–S3.

### Cell culture

Human urinary bladder transitional cell papilloma cell lines RT4 (HTB-2) and J82 (HTB-1) were purchased from the American Type Culture Collection (Manassas, VA USA). RT4 cells were cultured in McCoy’s 5A medium (16600082, Thermo Fisher Scientific, Waltham, MA, USA) supplemented with 10% fetal bovine serum (FBS; 10270106, Thermo Fisher Scientific, Waltham, MA, USA) and 1% antibiotics (15240-062, Thermo Fisher Scientific, Waltham, MA USA). J82 cells were maintained in EMEM (051-07615, Fujifilm Wako Pure Chemical Co., Osaka, Japan) supplemented with 10% FBS, 1% antibiotics, and 1% MEM nonessential amino acid solution (NEAA). All cell lines were authenticated using STR profiling (Additional file 2: Table S4). We routinely confirmed that these cell lines were negative for mycoplasma contamination using the e-Myco Mycoplasma PCR Detection Kit (25235, iNtRON Biotechnology, Inc., Korea).

### Plasmids and siRNAs

The lentiviral packaging plasmids pMD2.G (#12259) and psPAX2 (#12260) were obtained from Addgene (Watertown, MA, USA). To generate lentiviral vectors for conditional *PRELP* gene expression, a modified vector was constructed using Edit-R inducible lentiviral hEF1a-Blast-Cas9 nuclease plasmid DNA (CAS11229, GE Healthcare, Chicago, IL, USA) as the backbone. To generate a unique restriction site, the NheI restriction site was mutated immediately downstream of the hEF1 promoter region using the Gibson Assembly System (E2611, New England BioLabs, Ipswich, MA, USA). The expression construct of PRELP-myc was derived from the pCS2-PRELP-myc vector [20]. The PRELP-myc cDNA was PCR-amplified with the following primers: PRELP-myc_F_NheI: 5′-ACC CAA GCT GGC TAG CCA CCA TGA GGT CAC CCC TCT GCT G-3′, PRELP-myc_R_NotI: 5′-CAG CAC AGT GGC GGC CGC TCG AGT CTA GAC TAT AGT TCT AGA GGC TCG A-3′, and cloned into the modified Edit-R inducible lentiviral plasmid at NheI and NotI sites. All plasmids were verified by Sanger sequencing.

### Conditional protein expression of PRELP

A lentivirus transduction system was used to induce the conditional expression of PRELP. To produce lentiviruses, the viral vectors and packaging plasmids were co-transfected into 293T cells using Lipofectamine 3000 (L3000-008, Thermo Fisher Scientific, Waltham, MA, USA), according to the manufacturer’s instructions. After 48 h, the cell culture medium containing lentiviruses (for conditional PRELP-myc expression) was collected and filtered through a 0.45-μm filter. Target cell lines were plated in 24-well plates and cultured with a lentivirus-containing medium for 3 days in the absence of polybrene. PRELP-myc-expressing cells were selected with blasticidin S (10 μg/mL) (029-18701, Fujifilm Wako Pure Chemical Co., Osaka, Japan). Conditional expression was induced by the addition of 1 μg/mL of doxycycline (DOX) (D9891, Sigma-Aldrich, St. Louis, MO, USA).

### Western blotting

Cells were directly lysed with CelLyticM cell lysis reagent (C2978, Sigma-Aldrich, St. Louis, MO, USA) containing protease inhibitors (04693159001, Roche, Basel, Switzerland) or fractionated with Subcellular Protein Fractionation Kit (78840, Thermo Fisher Scientific, Waltham, MA, USA) according to manufacturer's instructions. Whole-cell lysates were passed through a 25-gauge needle ten times prior to centrifugation. Total protein concentration was measured using the Pierce 660 nm Protein Assay Reagent (22660, Thermo Fisher Scientific, Waltham, MA, USA). Whole-cell lysates mixed with Pierce Lane Marker Reducing Sample Buffer (39000, Thermo Fisher Scientific, Waltham, MA, USA) were boiled at 95 °C for 5 min, loaded into separate lanes on a sodium dodecyl sulfate (SDS) polyacrylamide gel (456-9034, Bio-Rad, California, USA), and then transferred to a nitrocellulose membrane (10600012, GE Healthcare, Chicago, IL, USA) following electrophoresis. After blocking with 5% skimmed milk (190-12865, Fujifilm Wako Pure Chemical Co., Osaka, Japan), the membranes were incubated with primary antibodies at 4 °C overnight. Protein bands were labeled with horseradish peroxidase (HRP)-conjugated secondary antibodies and visualized using ECL Prime western blotting detection reagent (RPN2236, GE Healthcare, Chicago, IL, USA) and ImageQuant LAS 4000 (GE Healthcare, Chicago, IL, USA). The primary antibodies used were anti-myc (sc-40; 1:1000, Santa Cruz Biotechnology, Dallas, TX, USA), mouse anti-PRELP antibody (#9; 1:1000, a gift from the Tsumoto laboratory), and anti-α-tubulin (CP06; 1:1000, Merck Millipore, Darmstadt, Germany). The secondary HRP-conjugated antibodies used were anti-mouse IgG (NA931; 1:5000, GE Healthcare, Chicago, IL, USA), and anti-rabbit IgG (NA934; 1:5000, GE Healthcare, Chicago, IL, USA). For the purpose of loading control, proteins on nitrocellulose membrane were visualized by SYPRO Ruby Protein Blot Stain kit (S-11791, Thermo Fisher Scientific, Waltham, MA, USA) according to manufacturer's instructions.

### Cell viability assay

For the cell proliferation assays, following overexpression of PRELP, the cells were plated in 96-well plates at 500 cells/well for RT4 and J82 cell lines. Culture media with the respective treatment reagents were replaced every 3 days. For experiments on the combined effects of cisplatin and HDACi, cells were plated in 96-well plates at 1000 and 1500 cells/well for RT4 and J82, respectively. After 24 h, the inhibitors were added at the indicated concentrations and incubated for 48 h. At the indicated time points, 10 μl of Cell Counting Kit-8 (343-07623, Dojindo, Kumamoto, Japan) reagent was added to each well. After 2 h of reaction, cell viability was analyzed by measuring the absorbance at 450 nm using Multiskan FC (Thermo Fisher Scientific, Waltham, MA, USA).

### Chemical compounds

The chemical compounds used in this study were SAHA (Suberoylanilide hydroxamic acid; SML0061, Sigma-Aldrich, St. Louis, MO, USA), trichostatin A (S1045, Selleck Chemicals, Houston, TX, USA), entinostat (S1053, Selleck Chemicals, Houston, TX, USA), 5-azacytidine (A2033, Tokyo Chemical Industry, Tokyo, Japan), UNC1999 (505052, Merck Millipore, Darmstadt, Germany), UNC0638 (382192, Merck Millipore, Darmstadt, Germany), BIX-01294 (S8006, Selleck Chemicals, Houston, TX, USA), C646 (S7152, Selleck Chemicals, Houston, TX, USA), SGC-CBP30 (S7256, Selleck Chemicals, Houston, TX, USA), LMK-235 (S7569, Selleck Chemicals, Houston, TX, USA), tacedinaline (C0621, Sigma-Aldrich, St. Louis, MO, USA), CAY10603 (S7596, Selleck Chemicals, Houston, TX, USA), RGFP966 (S7229, Selleck Chemicals, Houston, TX, USA), tubastatin A (S8049, Selleck Chemicals, Houston, TX, USA), bufexamac (HY-B0494, MedChemExpress, Monmouth Junction, NJ, USA), valproic acid (S3944, Selleck Chemicals, Houston, TX, USA), SIS17 (S6687, Selleck Chemicals, Houston, TX, USA), PCI-34051 (S2012, Selleck Chemicals, Houston, TX, USA), tasquinimod (S7617, Selleck Chemicals, Houston, TX, USA), TMP195 (2180, Axon Medchem, Groningen, Netherlands), santacruzamate A (S7595, Selleck Chemicals, Houston, TX, USA), paclitaxel (S1150, Selleck Chemicals, Houston, TX, USA) and cisplatin (S1166, Selleck Chemicals, Houston, TX, USA). EPZ011989 was obtained from Epizyme Inc., Cambridge, MA, USA.

### RT-PCR

Total RNA was extracted using QIAzol Lysis Reagent and RNeasy Plus Mini Kit (73404, Qiagen, Crawley, UK), and cDNA was synthesized using the PrimeScript RT Reagent Kit (RR037A, TaKaRa Bio, Shiga, Japan), according to the manufacturer’s instructions. Real-time PCR reactions were performed using TB Green Premix Ex Taq II (RR820A, TaKaRa Bio, Shiga, Japan) and the CFX96 Touch system (Bio-Rad, California, USA). *PRELP* mRNA levels were normalized to that of GAPDH (used as an internal control) using the ΔCq method. For quantitative real-time PCR, we used the following primers: PRELP_Forward: 5′-CTG TCC CAC AAC AGG ATC AG-3′; PRELP_Reverse, 5′-CAG GTC CGA GGA GAA GTC AT-3′; HDAC1_Forward: 5′-TTA TGG ACA AGG CCA CCC AAT G-3′; HDAC1_Reverse: 5′-ATT GGC TTT GTG AGG GCG ATA G-3′; HDAC2_Forward: 5′-ATG CTT GGA GGA GGT GGC TAC-3′; HDAC2_Reverse: 5′-TCT CAC AAT CAA GGG CAA CTG C-3′; GAPDH_Forward: 5′-GCA AAT TCC ATG GCA CCG TC-3′; GAPDH_Reverse: 5′-TCG CCC CAC TTG ATT TTG G-3′.

### Chromatin immunoprecipitation (ChIP)-qPCR

ChIP-qPCR was performed according to the manufacturer’s instructions (9003, Cell Signaling Technology, Denver, CO, USA), with minor modifications [58]. The cells were cross-linked with 1% formaldehyde for 10 min at room temperature, and cross-linking was quenched by the addition of a glycine solution. The fixed cells were washed three times with ice-cold phosphate-buffered saline (PBS). Nuclei preparation and chromatin digestion were performed according to the manufacturer’s instructions (9003, Cell Signaling Technology, Denver, CO, USA). Nuclei pellets were resuspended in ChIP buffer (50 mM Tris–HCl [pH 8.0], 150 mM NaCl, 1% Triton X-100, 0.5% IGEPAL CA-630, 5 mM EDTA, and protease inhibitor cocktail). Samples were sonicated using TOMY UR-21P (Tomy Seiko Co., Ltd., Tokyo, Japan) to generate DNA fragments of approximately 400–500 base pairs. Each antibody was added to the sheared chromatin and incubated in an ultrasonic water bath for 30 min at 4 °C. After centrifugation, the supernatants were incubated with FG Beads HM Protein G (TAB8848N3173, Tamagawa Seiki Co., Ltd., Nagano, Japan) for 30 min at 4 °C. Beads were washed with ChIP buffer and again with wash buffer (50 mM Tris–HCl [pH 8.0], 300 mM NaCl, 1% Triton X-100, 0.1% SDS, 0.1% Na-deoxycholate, and 5 mM EDTA) and LiCl buffer (50 mM Tris–HCl [pH 8.0], 250 mM LiCl, 1% Triton X-100, 0.5% Na-deoxycholate, and 5 mM EDTA). The immunoprecipitated chromatin was eluted, reverse-cross-linked with proteinase K, and purified using a PCR Clean-Up Mini Kit (FAPCK 001, FAVORGEN, Ping-Tung, Taiwan). Real-time PCR reactions were performed using TB Green Premix Ex Taq GC (RR071A, TaKaRa Bio, Shiga, Japan) and the CFX96 Touch system (Bio-Rad, California, USA). For ChIP-qPCR analysis, we used the following primers: PRELP_Forward_1: 5′-GGC CAG ACT TCT CCC TCT CT-3′; PRELP_Reverse_1: 5′-GAG TCT CAG GCT GGC ATA GG-3′; PRELP_Forward_2: 5′-GAA GGC AAG GCG ATT GTT AG-3′; PRELP_Reverse_2: 5′-TTG TTT GAC CCA TGT TTG GA-3′; PRELP_Forward_3: 5′-TCC AGG GTG AAC ATA GCA CA-3′; PRELP_Reverse_3: 5′-GTT CCT TGG GCC ATT CTT CT-3′. The following antibodies were used in this experiment: H3K9ac (#9649; 1:50, Cell Signaling Technology, Denver, CO, USA), H3K27ac (ab4729; 1 μg, Abcam, Cambridge, UK), H4K5ac (#8647; 1:25, Cell Signaling Technology, Denver, CO, USA), H4K12ac (#13944; 1:50, Cell Signaling Technology, Denver, CO, USA), H4K16ac (#13534; 1:50, Cell Signaling Technology, Denver, CO, USA), H2BK5ac (#12799; 1:50, Cell Signaling Technology, Denver, CO, USA), H2BK12ac (#9072; 1:50, Cell Signaling Technology, Denver, CO, USA), H2BK15ac (#9083; 1:50, Cell Signaling Technology, Denver, CO, USA), H3 (#4620; 1:50, Cell Signaling Technology, Denver, CO, USA), H2B (#12364; 1:50, Cell Signaling Technology, Denver, CO, USA), H4 (#14149; 1:50, Cell Signaling Technology, Denver, CO, USA).

### Synergism determination

To assess the combined effects of SAHA or entinostat with cisplatin, cell viability assay data were converted to a fraction of growth inhibition by each drug alone or by the drug combinations. Isobologram analysis was performed using CompuSyn software (v1, ComboSyn, Inc., Paramus, NJ, USA), which enabled the calculation of a combination index (CI) according to the Chou-Talalay CI-Isoblogram theory [59]. The CI indicates synergism at less than 1.0, antagonism at greater than 1.0, and additive at 1.0.

### Statistical analysis

Statistical analyses were performed using GraphPad Prism (v7, GraphPad Software, San Diego, CA, USA). *P* values are indicated in the figures and figure legends.

## Supplementary Information

**Additional file 1:** It includes supplementary Figs. 1–8 and supplementary raw data 1–3

**Additional file 2:** It includes supplementary tables 1–4. **Table S1**. Individual data for the correlation between PRELP mRNA expression and somatic mutations. **Table S2**. Individual data for the correlation between PRELP mRNA expression and copy number aberrations. **Table S3**. Individual data for the correlation between PRELP mRNA expression and DNA methylation. **Table S4**. Information about the certified cell lines. STR method was used for certification.

## Data Availability

The data supporting the findings of the present study are included in this article.
