# Supplementary material for: Repression of the PRELP gene is relieved by histone deacetylase inhibitors through acetylation of histone H2B lysine 5 in bladder cancer
Source: Clin Epigenetics. 2022 Nov 12;14:147. doi: 10.1186/s13148-022-01370-z (PMC9656081; doi:10.1186/s13148-022-01370-z)
Supplement: Supplementary file 1 — Additional file 1: It includes supplementary Figs. 1–8 and supplementary raw data 1–3 [file 13148_2022_1370_MOESM1_ESM.docx]

**Supplementary fig. 1**H2BK5 acetylation at the gene body of *PRELP* gene. **Top** Genomic positions (Region 3) of PCR amplified DNAs fragments are shown. **Bottom** ChIP-qPCR was performed using H2BK5ac antibodies at the *PRELP* gene body (Region 3) in RT4 cells treated with DMSO or 5 μM SAHA for 24 h. The ChIP enrichment (red bars) is normalized against the values obtained with the same antibodies in DMSO-treated control cells (black bars). Bars represent the mean ± SD of three technical replicates or mean ± standard error of the mean (SEM) of three biological replicates. Statistical analysis was performed using paired Student’s *t*-test. * *P* < 0.05.


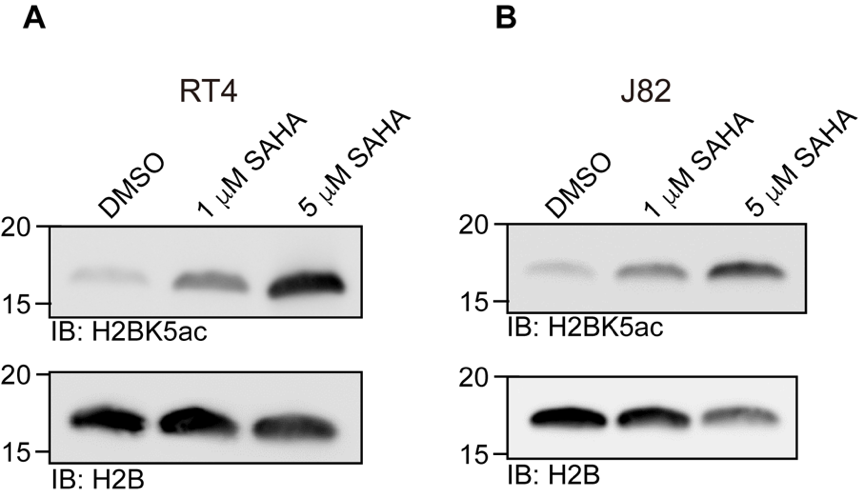


**Supplementary fig. 2** Acetylation of H2BK5 after SAHA treatment. RT4 **(A)** and J82 **(B)** cells were treated with the indicated concentrations of SAHA for 24 h. Acetylation of H2BK5 was analyzed using whole-cell extracts. The left side shows the protein size marker. H2B; loading control.


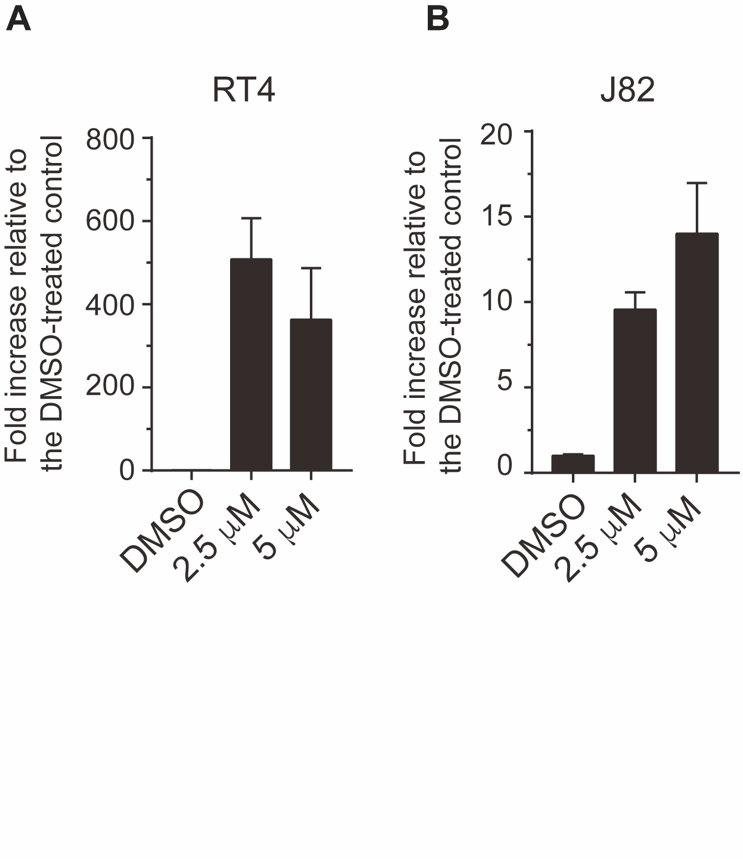


**Supplementary fig. 3** Restoration of PRELP gene expression by entinostat in bladder cancer cells. RT-qPCR analyses for PRELP (black bars) gene expression in RT4 **(A)** and J82 **(B)** cells. These cells were treated with either DMSO or the indicated concentrations of entinostat for 72 h. Data are presented as the mean ± standard deviation (SD) of three technical replicates. The y-axis shows the fold increase relative to DMSO-treated control.


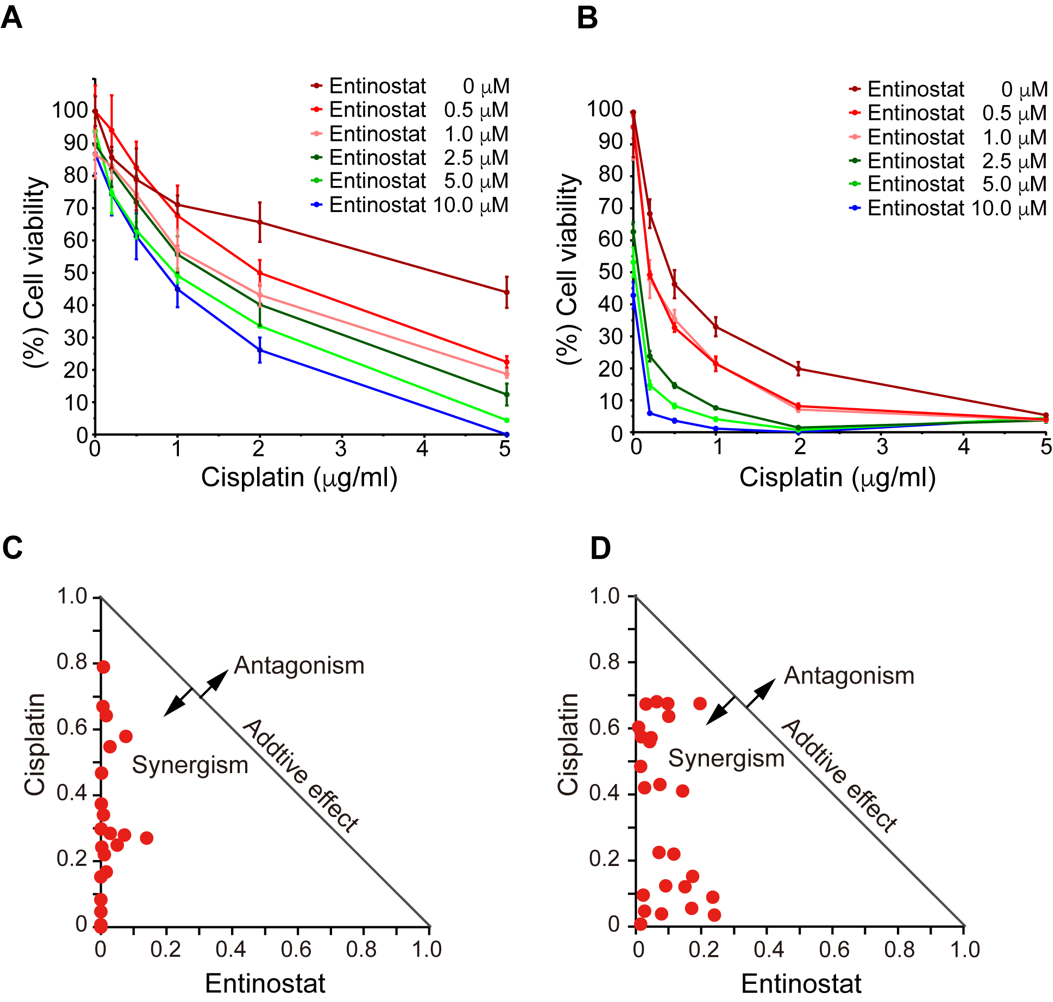


**Supplementary fig. 4** Combinatory effect of cisplatin and entinostat on viability of bladder cancer cells. RT4 **(A)** and J82 **(B)** cells were treated under the conditions shown in Figure 5, except that SAHA was replaced with entinostat. Normalized isobologram of RT4 **(C)** and J82 **(D)** show cisplatin and entinostat synergism.

**Supplementary fig. 5**Restoration of *PRELP* gene expression by various selective HDAC inhibitors in bladder cancer cells. RT-qPCR analyses of *PRELP* gene expression in RT4 (upper side) and J82 cells (bottom side). The cells were treated with either DMSO or increasing concentration (2.5 and 5 μM) of indicated compounds for 72 hours. Data are presented as the mean ± standard deviation (SD) of three technical replicates. The y-axis shows the fold increase relative to DMSO-treated control. The x-axis shows the name of each inhibitor. The red line indicates a fold increase of 1.

**Supplementary fig. 6** Combinatory effect of paclitaxel and SAHA on viability of bladder cancer cells. Cells were treated with paclitaxel alone or in combination with SAHA. Paclitaxel and SAHA were used at a concentration range of 0, 0.15625, 0.3125, 0.625, 1.25, 2.5 and 0, 0.5, 1.0, 2.5, 5.0, 10.0 μM, respectively. Cell viability of RT4 (left side) and J82 (right side) cells was evaluated using cell counting kit (CCK)-8 assays. We set the viability of cells with no inhibitor as 100% (n = 3).

**Supplementary fig. 7** Expression of *HDAC1, 2* during PRELP induction. RT-qPCR analyses for *HDAC1* (blue bars) and *HDAC2* (red bars) gene expression in RT4 (left side) and J82 (right side) cells during PRELP induction. Data are presented as the mean ± standard deviation (SD) of three technical replicates. The y-axis shows the *GAPDH* normalized fold increase relative to parental cells. n.s.; not significant.

**Supplementary fig. 8** Enrichment of PRELP protein at membrane fraction. Cytoplasmic (Cyto), membrane (Mem) and soluble nuclei (Sol nuclei) fractions were obtained by Subcellular protein fractionation kit **(See Methods)**. RT4 cells were treated with DMSO or 10 μM SAHA for 72 h. SYPRO Ruby protein blot stain was used as loading control. M; protein marker. PRELP protein is indicated by arrow. An asterisk indicates non-specific band.


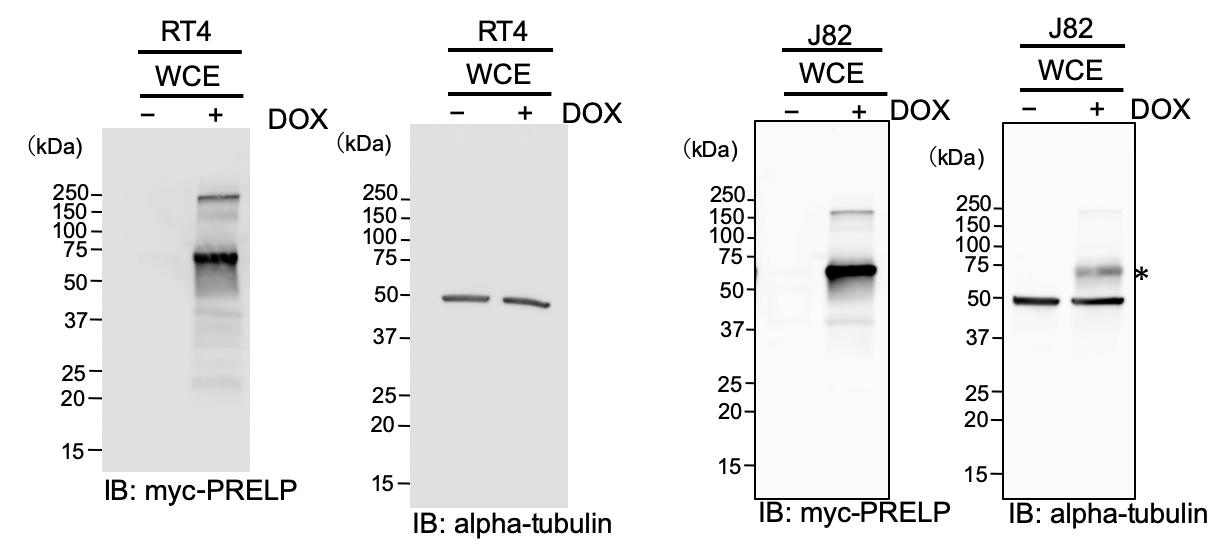


**Supplementary raw data 1** Uncropped immunoblot data shown in fig. 2. Asterisks indicate insufficiently stripped myc-PRELP.

**
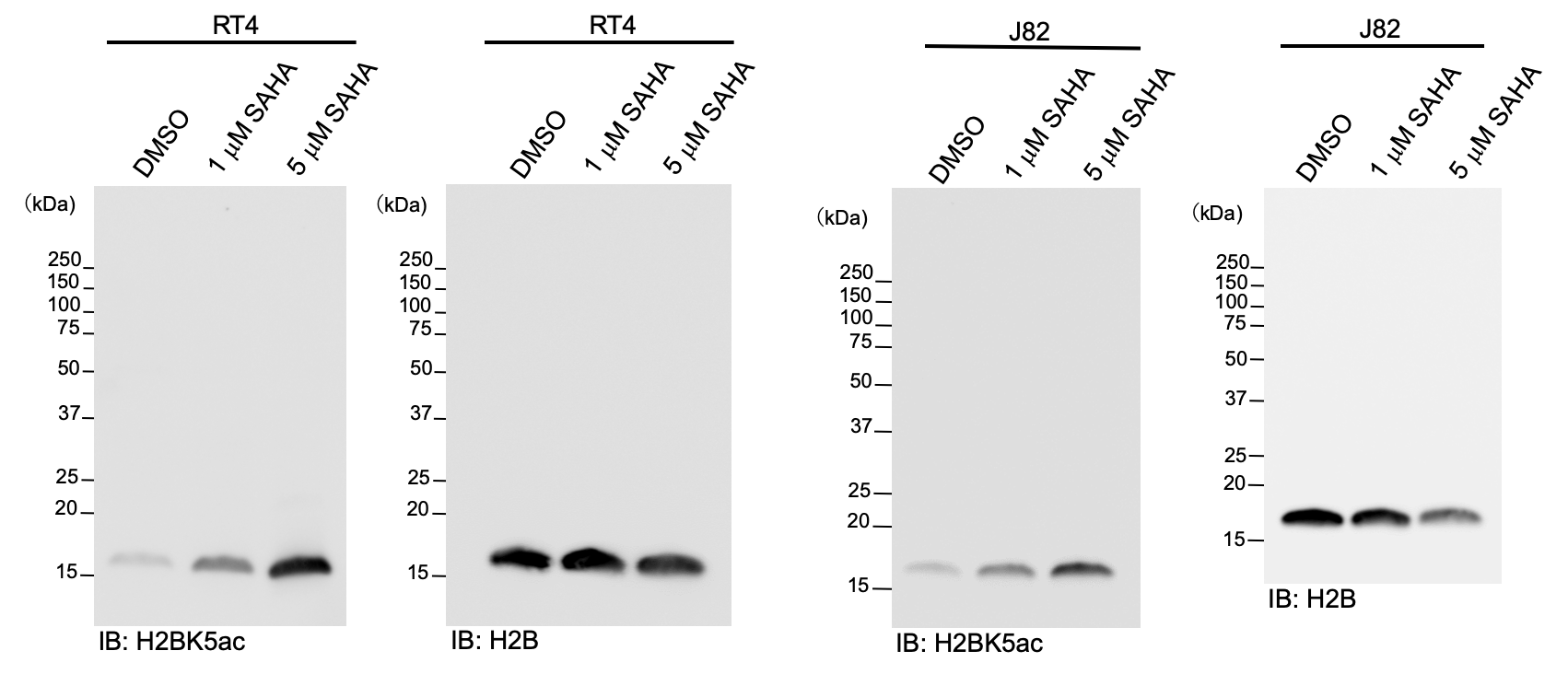
**

**Supplementary raw data 2** Uncropped immunoblot data shown in Supplemental fig. 2.

**Supplementary raw data 3** Uncropped immunoblot data shown in Supplemental fig. 8. Asterisks indicate non-specific bands.
